# Practice testing enhances learning but not attitude change from persuasive texts

**DOI:** 10.1038/s41598-025-20874-1

**Published:** 2025-09-25

**Authors:** Elena M. Galeano Weber, Lisa Lehnen, Doug Lombardi, Garvin Brod

**Affiliations:** 1 DIPF|Leibniz Institute for Research and Information in Education, and IDeA Research Center for Individual Development and Adaptive Education of Children at Risk, Rostocker Str. 6, 60323 Frankfurt am Main, Germany; 2 Senckenberg Biodiversity and Climate Research Centre, Frankfurt am Main, Germany; 3 University of Maryland, College Park, USA; 4 Goethe University, Frankfurt, Germany

**Keywords:** Persuasive texts, Practice testing, Knowledge acquisition, Attitude change, Retrieval Practice, Pretesting, Psychology, Human behaviour

## Abstract

We examined whether practice testing enhances learning from persuasive texts and influences attitude change. In two online experiments, participants read texts on biodiversity loss (Study 1, *n* = 454) or wolf recolonization (Study 2, *n* = 400) and were assigned to one of three conditions: pretesting (guessing before reading), posttesting (retrieving after reading), or no-testing. Both testing conditions improved knowledge retention compared to no-testing, with no consistent differences between pre- and posttesting. Across all conditions, attitudes shifted in the expected direction—biodiversity loss was perceived as more severe, and wolf recolonization more positively after reading. However, neither testing condition led to greater attitude change than no-testing. A systematic association between final knowledge and attitude emerged only in the pretesting group, suggesting that generating responses before learning may have promoted attitude change in some individuals. These findings demonstrate that practice testing enhances knowledge acquisition from persuasive texts but does not reliably amplify attitude change. This suggests that while retrieval-based learning strengthens memory for persuasive content, additional factors may be required to translate knowledge gains into belief revision. Our results highlight the complex relationship between knowledge and attitudes and contribute to understanding the role of test-enhanced learning in persuasion.

**Supplementary Information:**

The online version contains supplementary material available at 10.1038/s41598-025-20874-1.

## Introduction

Human memory is highly adaptable, allowing for both long-term information storage and the integration of new concepts and beliefs^1,2^. However, learning is not just about storing information—it also involves reasoning, evaluating evidence, and adapting beliefs in response to new input^3,4^. One of the most well-documented strategies for enhancing long-term memory is practice testing, which engages learners in test-like situations to reinforce knowledge^5,6^. This strategy includes pretesting, where learners attempt to generate responses before encountering correct information^7^, and posttesting (also termed as retrieval practice), where they recall studied material from memory^6^. While practice testing is well established as a strategy for improving factual learning, its role in shaping attitudes and facilitating learning from persuasive texts remains largely unexplored.

Attitude formation and change are central to persuasion research, which explores how individuals process and incorporate new information into their belief systems^8–10^. According to the Elaboration Likelihood Model (ELM)^11^, attitude change is strongest when individuals engage in deep, effortful cognitive processing rather than relying on superficial cues. Practice testing may encourage such deeper engagement, but its role in persuasion has received little attention. Pretesting may enhance attentional focus on key arguments^12,13^, increasing engagement with persuasive content and making individuals more receptive to attitude change. In contrast, retrieval practice reinforces memory for the presented arguments, which may strengthen existing attitudes or, when new information is integrated, promote attitude change. Both strategies may thus influence persuasion not only by affecting the amount of information remembered but also by shaping how new information interacts with existing knowledge.

This study examines whether pretesting and retrieval practice influence both knowledge acquisition and attitude change when learning from persuasive texts. Specifically, we test whether pretesting enhances cognitive engagement with persuasive arguments, making individuals more open to attitude change, and whether posttesting strengthens memory for persuasive content, reinforcing attitude-consistent knowledge. To better understand how knowledge acquisition and attitude formation interact, we first outline the cognitive mechanisms that may underlie this relationship.

### Knowledge, attitudes, and their interplay

Knowledge refers to factual representations, while attitudes involve evaluative judgments that integrate cognitive, affective, and behavioral components^14,15^. Although the strength and accessibility of knowledge can shape attitudes^16^, belief systems, reasoning processes, and motivation also influences how new information is interpreted^3,4^. The relationship is bidirectional: new knowledge can shift attitudes, and pre-existing attitudes can guide how knowledge is processed^17,18^. Cognitive dissonance theory^17^ holds that conflicts between knowledge and attitudes prompt belief adjustment to reduce discomfort, while the Elaboration Likelihood Model^11^ suggests that deep cognitive processing (central-route processing) makes attitude change more likely.

### Learning from persuasive texts and practice testing

Practice testing may foster deep processing and active integration of the new, conflicting information by co-activating prior knowledge and new information (see the Knowledge Revision Components framework)^19^. For example, guessing “10% of species are threatened” but getting the feedback it is “25%” co-activates prior and new information. As the amount and interconnectedness of the new information that it is 25% increases during reading of the text, it increasingly dominates the learner’s mental network, reducing interference from prior information. Because attitudes rest on underlying beliefs, such mechanisms—and others, such as elaborative processing or heightened prediction error monitoring—could plausibly support lasting attitude change.

These mechanisms may be particularly relevant for persuasive texts, which aim to challenge existing beliefs and introduce new perspectives, fostering both knowledge acquisition and attitude change ^9,10^. Such texts can increase acceptance of scientific explanations^20^ and alter attitudes on controversial issues such as climate change^21^, but their impact can depend on the depth of cognitive engagement.

Practice testing—which includes pretesting (answering questions before learning) and posttesting (retrieval practice)—has been shown to enhance long-term retention, comprehension, and information integration^5,22–27^. Though typically studied in the context of factual learning, it may also affect persuasion by influencing how argumentative content is processed and stored. Pretesting, also known as errorful generation, aligns with prediction-based learning: by making knowledge gaps salient, it can heighten attention to key arguments and trigger coactivation of relevant prior knowledge, facilitating integration of new content^13,19,28–31^. Posttesting or retrieval practice, in contrast, consolidate learning by strengthening retrieval cues and conceptual understanding^30,32,33^. Both are most effective with immediate corrective feedback to ensure the reinforcement of accurate knowledge^34^.

### Present study

Despite extensive research on practice testing and memory, its role in persuasion and attitude change remains unclear. Prior work suggests that pretesting can heighten engagement with new information^7^, while retrieval practice strengthens long-term retention and conceptual understanding^32^. Yet, little is known about whether these strategies, when applied to persuasive texts, shape attitudes as well as knowledge.

We addressed this question in two online experiments on environmental conservation topics—biodiversity loss (Study 1, preregistered) and wolf recolonization in Europe (Study 2, conceptual replication)—where both knowledge and attitudes influence behavior^35,36^. Participants were randomly assigned to pretesting, posttesting, or no-testing control conditions. Pretesting involved answering numerical fact-based questions before reading the persuasive text (interim knowledge test); posttesting involved answering the same questions after reading; both testing conditions included immediate corrective feedback, while the control group read without interim testing. All participants completed pre- and post-reading attitude measures, a working memory task to control for potential differences in cognitive capacity, and a final knowledge test without feedback (Table 1).

**Table 1:** Experimental design.

Study group			
	No-testing	Pretesting	Posttesting
1	Pre-attitude questions (a)	Pre-attitude questions (a)	Pre-attitude questions (a)
2	Text reading (c)	Interim knowledge test (b)	Text reading (c)
3	WM updating task (d)	Text reading (c)	WM updating task (d)
4	Post-attitude questions (e)	WM updating task (d)	Interim knowledge test (b)
5	Final knowledge test (f)	Post-attitude questions (e)	Post-attitude questions (e)
6	/	Final knowledge test (f)	Final knowledge test (f)

WM: Working Memory.

Based on prior evidence that retrieval practice enhances memory accuracy^6,37^, and that pretesting increases cognitive engagement with new content^7,12,26^, we predicted that the no-testing condition would yield lower memory accuracy than either posttesting (1a) or pretesting (1b), with no significant difference between the two testing conditions (1c). Pretesting heightens attention to key arguments^13,30^, and promotes integration of new with prior knowledge^19^. We therefore hypothesized that pretesting would produce the strongest attitude shifts, whereas posttesting would primarily reinforce accurate knowledge with some potential for updating (2a,b). All hypotheses (1a–c, 2a,b) were preregistered for Study 1 (https://osf.io/a4ghd). Finally, consistent with evidence that greater knowledge accuracy predicts belief and attitude change ^16,18^, we tested whether higher accuracy—particularly in pretesting—would be linked to greater attitude change. Cognitive dissonance theory^17^ suggests that awareness of knowledge gaps creates cognitive conflict, motivating deeper processing of persuasive arguments. By making these knowledge gaps more salient, pretesting may foster integration of new information with prior beliefs^7,12,19,26^, whereas posttesting (retrieval practice) tends to reinforce argument-consistent content^6^ without necessarily prompting belief revision (3). By addressing these questions, this study aims to clarify the role of practice testing in both knowledge acquisition and persuasion, contributing to scientific communication, education, and misinformation resistance.

## Results

**Fig. 1:**
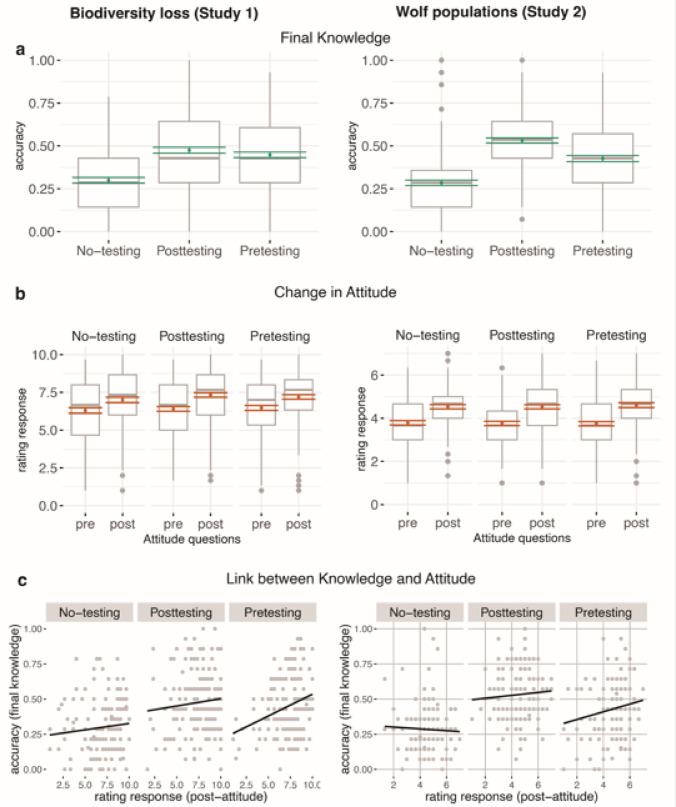
Results from Study 1 and Study 2 are plotted on the left and right side, respectively (**a**) Plotted are the distributions of response accuracy in the final knowledge test showing the means (green dots) with standard errors under no-testing, posttesting, and pretesting conditions. (**b**) Distributions of rating responses from pre- and post-attitude questions, i.e., before and after the learnings sessions, respectively, for each study condition. Dots represent the means and bars the standard errors under each condition. (**c**) Scatterplots with regression lines showing the relationship between the rating response from the post-attitude questions (after the learning session) and response accuracy of the final knowledge test for each study condition. Each dot corresponds to an individual.

Figure 1 provides an overview of the main results from Study 1 (left panels) and Study 2 (right panels). It illustrates group-level distributions of response accuracy across study conditions (a), shifts in attitude ratings before and after the learning sessions (b), and the relationship between post-attitude ratings and final test performance at the individual level (c).

## Study 1

### Effects of practice testing on changes in knowledge

The interim knowledge test yielded low accuracy close to zero under the pretesting condition, as expected, showing that participants knew almost none of the facts before reading the text (detailed descriptive statistics can be found in Table 2). In the posttesting condition, accuracy in the first knowledge test, i.e., provided immediately after reading the text, was significantly higher than under pretesting (*t*_(*df* = 314)_ = 14.301, *p* < 0.0001; *partial η*^*2*^ = 0.39), indicating a systematic effect of reading the text on factual knowledge.

**Table 2:** Summary statistics of memory accuracy in the interim and final knowledge tests and pre- and post-attitude rating responses for each study group of Study 1 and Study 2.

		Biodiversity loss (Study 1)				Wolf populations (Study 2)			
	Study group	*mean*	*sd*	*min*	*max*	*mean*	*sd*	*min*	*max*
Interim Knowledge	Pretesting	0.04	0.06	0	0.29	0.04	0.05	0	0.21
	Posttesting	0.28	0.19	0	1	0.29	0.18	0	0.93
Final Knowledge	Pretesting	0.45	0.2	0	0.93	0.43	0.2	0	0.93
	Posttesting	0.47	0.22	0	1	0.53	0.18	0.07	1
	No-testing	0.3	0.19	0	0.79	0.28	0.18	0	1
Pre-attitude	Pretesting	6.47	2.02	1	9.67	3.75	1.14	1	6.67
	Posttesting	6.4	1.96	1.67	9.67	3.76	1.14	1	6.33
	No-testing	6.3	2.17	1	9.67	3.79	1.11	1	6.33
Post-attitude	Pretesting	7.2	1.85	1	10	4.61	1.26	1	7
	Posttesting	7.33	1.93	1.67	10	4.53	1.17	1	7
	No-testing	7	2.1	1	10	4.53	1.14	1.33	7

Of most interest were the results of the final knowledge test, which also included data from the no-testing group. Here, both pre- and posttesting led to significantly higher accuracies than no-testing with moderate effect sizes (Model H1: Pretesting > No-testing *t*_(d*f* =451)_ = 6.157, *p* < 0.0001, *partial η*^*2*^ = 0.11; Posttesting > No-testing: *t*_(*df* = 451)_ = 7.356, *p* < 0.0001, *partial η*^*2*^ = 0.08), and did not significantly differ from each other (Model H1: Pretesting ~ Posttesting: *t*_*(df* = 451)_ = 1.180, *p* = 0.465) (Fig. 1a). Thus, participants in the no-testing group remembered significantly less of the facts in the text than participants under the testing conditions. Whether additional testing was performed prior to reading the text (i.e., pretesting) or afterwards (i.e., posttesting) did not systematically matter. These results supported our preregistered hypotheses (H1a-c).

#### Effects of practice testing on changes in attitude

Figure 1b shows the distributions of rating response measure of each study group for the first and second round of attitude questions, which were presented at the beginning and at the end of the experiment, respectively (cf., Table 2).

Results from secondary analyses that are not related to core hypotheses revealed that biodiversity loss was perceived as more threatening from pre- to post ratings within all three study groups as shown by a significant main effect of pre-/post-attitude (Model 1a: *t*_(*df* = 453)_ = 11.498, *p* < 0.0001, *partial η*^2^ = 0.23). Study groups did not significantly differ in their overall amount of rating response (Model 1a: No-testing ~ Posttesting *t*_(*df* = 451)_ = -1.078, *p* = 0.528; No-testing ~ Pretesting* t*_(*df* = 451)_ = -0.850, *p* = 0.672; Posttesting ~ Pretesting *t*_(*df* = 451)_ =  −0.228, *p* = 0.971), and we found no significant interaction effects for study groups and pre-/post attitude ratings suggesting that changes from pre- to post ratings were similar between groups (Model 1b: No-testing ~ Posttesting* t*_(*df* =451)_ = 1.331, *p* = 0.184; No-testing ~ Pretesting *t*_(*df* =451)_ = 0.152, *p* = 0.879).

These findings were supported by the planned comparisons. Neither did posttesting lead to significantly greater attitude changes than no-testing (Model H2: *t*_(*df* = 451)_ = 1.331, *p* = 0.184), nor did pretesting lead to significantly greater attitude changes than posttesting (Model H2: *t*_*(df* = 451)_ =  −1.214, *p* = 0.225). The non-significant results were further supported by the posterior mean estimates and 95% credible intervals of an equivalent Bayesian analysis on the effect of study group on attitude change (Pretesting ~ Posttesting: *β* =  −0.21, 95% *CI*[-0.53, 0.12]; Posttesting ~ No-testing: *β* = 0.23, 95% *C*I[ −0.09, 0.45]). These results indicate that study groups showed no meaningful differences in attitude change. Hence, our pre-registered hypotheses (H2a, H2b) were not supported. While the loss of biodiversity was perceived as more threatening in the attitude posttest compared to pretest, this effect did not significantly differ by condition.

#### Links between attitude and knowledge

Even in the absence of significant mean group differences in attitude change, the differences in final knowledge between conditions could still be differentially linked to participants’ attitude. We therefore tested whether participants’ performance in the final knowledge test was associated with their post attitude ratings separately for each study condition. For the pretesting group, we observed a significant positive association between final knowledge and post-attitude with a moderate effect size (Model 2c: *t*_(*df* = 153)_ = 3.703, *p* < 0.0001, *partial η*^*2*^ = 0.08) such that participants who showed higher accuracy in the final knowledge test rated the loss in biodiversity as more severe after the learning session than participants with lower accuracy. No significant effect was found for posttesting or no-testing groups (Model 2a, No-testing: *t*_(*df* = 136)_ = 1.151, *p* = 0.252; Model 2b, Posttesting: *t*_(*df* = 159)_ = 1.157, *p* = 0.249). The non-significant results were supported by the results of Bayesian model equivalents (No-testing: *β* = 1.08, 95% *CI*[ −0.86, 3.05]; Posttesting: *β* = 0.80, 95% *CI*[ −0.54, 2.13]).

In addition to our main analyses, the results of several control analyses are presented in the following sections.

#### Baseline performances and test reliabilities of attitude and knowledge items

Groups did not significantly differ in their pre-attitude ratings (Posttesting ~ No-testing: *t*_(*df* =451)_ = -0.423, *p* = 0.906; Pretesting ~ No-testing: *t*_(*df* =451)_ =  −0.708, *p* = 0.759; Posttesting ~ Pretesting: *t*_(*df* =451)_ =  −0.300, *p* = 0.951) suggesting that they had similar baseline rating measures. Internal consistency of the three attitude items was satisfactory as shown by Cronbach’s Alphas of 0.84 and 0.87 of the first and second attitude questions, respectively. The fourteen knowledge items showed acceptable measures of reliability with Cronbach’s Alphas of 0.74 and 0.79 of the first/interim and second/final knowledge test, respectively.

#### Effects of load and study group on working memory

Participants also completed a preregistered numerical working memory (WM) updating task. In each trial, three (Load 3) or four (Load 4) one-digit numbers were presented simultaneously in separate cells of a grid, then updated through a sequence of arithmetic operations—four updates for load 3 and five for load 4—before recalling the final values. Participants showed lower WM accuracy under load 4 than 3 (Load 4 < Load 3: *t*_*(df* = 451)_ =  −13.534, *p* < 0.0001, *partial η*^*2*^ = 0.29). Study groups did not significantly differ in performance (Pretesting ~ No-testing: *t*_(*df* = 451)_ =  −0.093, *p* = 0.995: Posttesting ~ No-testing: *t*_(*df* = 451)_ = 0.081, *p* = 0.996; Pretesting ~ Posttesing: *t*_(*df* = 451)_ = 0.013,* p* = 0.999), and study groups did also not significantly interact with load manipulation (Posttesing: *t*_(*df* =449)_ = 1.030, *p* = 0.303; Pretesing: *t*_(*df* =449)_ = 0.724, *p* = 0.469).

#### Links between working memory and knowledge gain

Given evidence that working memory can moderate testing effects, we tested whether WM accuracy (aggregated across Load 3 and Load 4) predicted final knowledge test performance. Linear mixed-effects models were specified with accuracy as the dependent variable, WM accuracy as the predictor, and subject as a random intercept, estimated separately for each study group. Higher WM accuracy was significantly linked to greater knowledge gain in both pre- and posttesting groups (Pretesting: *t*_(*df* = 152)_ = 5.035, *p* < 0.01 ; Posttesting: *t*_(*df* = 159)_ = 5.335, *p* < 0.01), but not in the no-testing group (*t*_(*df* = 135)_ = 1.695, *p* = 0.092).

#### Knowledge–attitude link under pretesting, controlling for WM and prior knowledge

The positive association between knowledge gain and attitude change under pretesting may have been influenced by participants’ prior knowledge or cognitive ability. To examine this, we estimated two linear mixed-effects models with subject as a random intercept within the pretesting group: one predicting post attitude ratings from final knowledge accuracy and WM updating accuracy (aggregated across Load 3 and Load 4), and another predicting post attitude ratings from final knowledge accuracy and interim knowledge test accuracy, used as a proxy for prior knowledge of the conservation topic. Prior knowledge did not significantly correlate with final attitude (*t*_(*df* = 152 )_ =  −1.035, *p* = 0.302), and the association between final knowledge and attitude remained significant when prior knowledge was controlled for (*t*_(*df* = 152)_ = 3.846, *p* < 0.001). Similarly, WM accuracy did not significantly affect attitude ratings (*t*_(*df* = 152 )_ =  −0.304, *p* = 0.761), and final knowledge continued to show a significant positive association with attitude when WM was controlled for (*t*_(*df* = 152 )_ = 3.541, *p* < 0.001).

#### Text reading times

Participants of the pretesting group tended to spend less time on the text per slide than participants of the no-testing group (*M, SD*_pretesting_ = 16.6, 10.9 s., *M, SD*_no-testing_ = 20.0, 14.9 s.; *t*_(*df* = 451)_ =  −2.246, *p* = 0.065); reading duration did not significantly differ between pretesting and posttesting (*M, SD*_posttesting_ = 19.60 12.67 s.; *t*_(*df* = 451)_ = 2.042, *p* = 0.104), nor under posttesting and no-testing (*t*_(*df* = 451)_ = 0.286, *p* = 0.956). When exploring the link between reading times and final knowledge, we found a significant positive association under pretesting with a small effect size (*t*_(*df* = 153)_ = 2.062, *p* = 0.041, partial η^2^ = 0.03); we observed no significant effects under posttesting (*t*_*(df* = *159)*_ = 1.932, *p* = 0.055) nor no-testing (*t*_(*df* = 136)_ = 1.233,* p* = 0.219).

### Study 2

Study 2 served as a conceptual replication of Study 1, with our main motivation being to examine a more controversial topic about which participants perhaps felt more strongly. We therefore used a persuasive text on wolf recolonization in Europe—a topic that has sparked considerable debate due to conflicts between conservation goals and rural interests, and which thus provides a stronger test case for attitude change. A total of 400 participants were randomly assigned to pretesting, posttesting, or no-testing conditions. Procedures closely followed those of Study 1, with adaptations to the text content and corresponding knowledge and attitude measures to reflect the new topic.

#### Testing effects on changes in knowledge

As in study 1, the interim knowledge test yielded low accuracy (close to zero) under the pretesting condition showing that participants had little prior knowledge before reading the text (cf. Table 2). Response accuracy under posttesting was significantly higher than under pretesting in the interim knowledge test (*t*_(*df* = 274)_ = 14.760, *p* < 0.0001) with an effect size of *partial η*^*2*^ = 0.44, indicating a large effect of reading the text on factual knowledge.

The mean accuracies in the final knowledge tests were 0.43 and 0.53 under pre- and posttesting, respectively, suggesting that the tests were quite challenging for most participants and showed no ceiling effects in both groups (Table 2). Both pre- and posttesting led to significantly higher accuracies than no-testing with medium to large effect sizes (Model H1: Pretesting > No-testing *t*_(*df* = 397)_ = 6.065, *p* < 0.0001, *partial η*^*2*^ = 0.08; Posttesting > No-testing: *t*_(*df* =397)_ = 10.804, *p* < 0.0001, *partial η*^*2*^ = 0.23 ). While we found no significant difference between the testing conditions in study 1, data from Study 2 revealed significantly higher accuracy in the final knowledge under posttesting compared to pretesting with a small effect size (Model H1: Posttesting > Pretesting: *t*_(*df* = 397)_ = 4.690, *p* < 0.0001, *partial η*^*2*^ = 0.05) (Fig. 1a, right side).

#### Testing effects on attitude changes

Figure 1b shows the distributions of rating response measure of each study group for the pre- and post-attitude questions which were presented before and after the experiment, respectively (see Table 2 for descriptive statistics).

Results from secondary analyses revealed that attitude towards wolves became significantly more positive from pre- to post-attitude ratings within all three study groups as shown by a significant main effect of pre-/post attitude with a large effect size (Model 1a: *t*_(*df* = 399)_ = 17.458, *p* < 0.0001, *partial η*^*2*^ = 0.43). Study groups did not significantly differ in their overall ratings (Model 1a: No-testing ~ Posttesting *t*_(*df* = 397)_ = 0.156, *p* = 0.987; No-testing ~ Pretesting *t*_(*df* = 397)_ =  −0.034, *p* = 0.999; Posttesting ~ Pretesting *t*_(*df* = 397)_ =  −0.194, *p* = 0.980), and we found no significant interaction effects for study groups and pre-/post attitude ratings suggesting that changes from pre- to post ratings were similar between groups (Model 1b: Posttesting *t*_*(*df = 397)_ = 0.280 , *p* = 0.779; Pretesting *t*_*(df* = 397)_ = 1.048, *p* = 0.295).

These findings were supported by the planned comparison analysis. Neither did posttesting lead to greater attitude changes than no-testing (Model H2: *t*_(*df* = 397)_ = 0.280, *p* = 0.779), nor did pretesting lead to greater attitude changes than posttesting (Model H2: t_(*df* = 397)_ = 0.803, *p* = 0.422). The non-significant results were further supported by an equivalent Bayesian analysis on the effect of study group on attitude change (Pretesting ~ Posttesting: *β* = 0.09 ,95% *CI*[ −0.12, 0.30]; Posttesting ~ No-testing: *β* = 0.02, 95% *CI*[-0.20, 0.24]).

In sum, findings are in line with those of study 1: while wolf populations were perceived as more positive in the attitude posttest compared to pretest, this effect did not systematically differ by condition.

#### Links between attitude and knowledge

As in study 1, we ran linear mixed effects models separately for each study condition to test whether the differential changes in knowledge between condition was associated to the differential changes in attitude. For the pretesting group, we observed a tendency for a positive association between final knowledge and post-attitude with a small effect size (Model 2c: *t*_(*df* = 130)_ = 1.966, *p* = 0.051, *partial η*^*2*^ = 0.03) such that participants who showed higher accuracy in the final knowledge test tended to rate wolves as more positive after the learning session than participants with lower accuracy. No significant effects were also found for posttesting or no-testing groups (Model 2a, No-testing: *t*_(*df* = 122)_ =  −0.467, *p* = 0.641; Model 2b, Posttesting: *t*_(*df* = 142 )_ = 0.796, *p* = 0.427). The non-significant results were supported by the results of Bayesian model equivalents (No-testing: *β* =  −0.30, 95% *CI*[ −1.60, 0.84]; Posttesting: *β* = 0.44, 95% *CI*[ −0.65, 1.54]; Pretesting: *β* = 1.05, 95% *CI*[ −0.06, 2.11]).

Table 3 summarizes and compares the main results of both studies by showing the effect sizes of each test (Table 3). We again performed several control analyses, which are presented below.

**Table 3:** Summary of linear mixed effects models and results of Study 1 in comparison to Study 2 showing effect sizes.

	Test	Effect size (partial η^2^)	
		Study 1	Study 2
Final knowledge	Contrast coding: Pretesting > No-testing Posttesting > No-testing Posttesting ~ Pretesting	**Pretesting > No-testing: 0.11** **Posttesting > No-testing: 0.08** *Posttesting* ~ *Pretesting: n.s*	**Pretesting > No-testing: 0.08** **Posttesting > No-testing: 0.23** ***Posttesting* > *Pretesting: 0.05***
Attitude	Main effect of Pre-/Post Attitude Main effect of Study group Study group x Pre-/Post Attitude Contrast coding: No-testing < Posttesting Posttesting < Pretesting	**Post- > Pre-Attitude: 0.23** Pretesting ~ No-testing: n.s Posttesting ~ No-testing: n.s Posttesting ~ Pretesting: n.s Pre-/Posttesting x Post-Attitude: n.s No-testing ~ Posttesting: n.s Posttesting ~ Pretesting: n.s	**Post- > Pre-Attitude: 0.43** Pretesting ~ No-testing: n.s Posttesting ~ No-testing: n.s Posttesting ~ Pretesting: n.s Pre-/Posttesting x Post-Attitude: n.s No-testing ~ Posttesting: n.s Posttesting ~ Pretesting: n.s
Final Knowledge and Attitude	Pretesting Posttesting No-testing	**Post-attitude: 0.08** Post-attitude: n.s Post-attitude: n.s	**Post-attitude: 0.03** Post-attitude: n.s Post-attitude: n.s

Significant results are in bold text, whereas normal (roman) font indicates non-significant results. Cases where results were inconsistent between studies (i.e., significant in one study, but not the other), are highlighted in italics.

#### Baseline performances and test reliabilities of attitude and knowledge items

Groups did not significantly differ in their pre-attitude ratings (No-testing ~ Posttesting: *t*_(*df* = 397)_ = 0.241, *p* = 0.968; No-testing ~ Pretesting: *t*_(*df* = 397)_ = 0.321, *p* = 0.945; Posttesting ~ Pretesting: *t*_(*df* = 397)_ = 0.088, *p* = 0.996) suggesting that they had similar baseline rating measures. Internal consistency of the three attitude items was satisfactory as shown by Cronbach’s Alphas of 0.86 and 0.89 of the first and second attitude questions, respectively. The 14 knowledge items showed acceptable measures of reliability with Cronbach’s Alphas of 0.70 and 0.76 of the first and second/final knowledge test, respectively.

#### Effects of load and study group on working memory

Results showed significant main effects of load (Load 4 < Load 3; *t*_*(df* = *398)*_ =  −13.429, *p* < 0.0001, *partial* ${\eta }^{2}$= 0.31) and study group whereby participants of the pretesting group showed significantly lower WM accuracy compared to participants of the no-testing group (Pretesting < No-testing: *t*_*(df* =*397 )*_ = 2.497, *p* = 0.034; *partial* ${\eta }^{2}$= 0.02). There were no further significant differences in WM accuracy between study groups (Pretesting ~ Posttesting: *t*_*(df* = *397)*_ = 1.695, *p* = 0.208; No-testing ~ Posttesting: *t*_*(df* = *397)*_ = 0.881, *p* = 0.653), and study group did not interact with load (Posttesting: *t*_*(df* = *396)*_ = 0.300, *p* = 0.764; Pretesting: *t*_*(df* = *396)*_ = 0.172, *p* = 0.863).

#### Links between working memory and knowledge gain

For each study group, we tested whether higher knowledge gain could be predicted by higher WM accuracy (aggregated across Load 3 and Load 4) using linear mixed-effects models with subject as a random intercept. Working memory accuracy was significantly linked to greater knowledge gain in both pre- and posttesting groups (Pretesting: *t*_(*df* = 130)_ = 4.644, *p* < 0.01 ; Posttesting: *t*_(*df* = 141)_ = 2.316 , *p* = 0.022), but not in the no-testing group (*t*_(*df* = 122)_ = 1.755, *p* = 0.082).

#### Knowledge–attitude link under pretesting, controlling for WM and prior knowledge

To test whether the knowledge–attitude link under pretesting was influenced by prior knowledge and/or WM, we ran two linear mixed-effects models with subject as a random intercept within the pretesting group. Prior knowledge (interim test accuracy) was not significantly correlated with post attitude ratings (*t*_(*df* = 129)_ =  −0.417, *p* = 0.677), and the positive link between final knowledge and attitude remained marginally significant when prior knowledge was controlled for (*t*_(*df* = 129)_ = 1.960, *p* = 0.052). Similarly, WM accuracy did not significantly affect attitude ratings (*t*_(*df* = 129 )_ = 1.143, *p* = 0.255); the association between final knowledge and attitude was no longer significant when WM was controlled for (*t*_(*df* = 129 )_ = 1.392, *p* = 0.166).

#### Text reading times

Mean reading times per slide were comparable to Study 1 (*M, SD*_pretesting_ = 17.0, 8.6, sec., *M, SD*_no-testing_ = 18.1, 11.0 s.; *M, SD*_posttesting_ = 18.2, 12.1 s.), but there was no significant effect of study condition (Pretesting ~ No-testing *t*_(df = 397)_ = 0.846 , *p* = 0.675; Posttesting ~ No-testing *t*_(df = 397)_ =  −0.036, *p* = 0.999; Pretesting ~ Posttesting *t*_(df = 397)_ = 0.914,* p* = 0.632) on reading duration. There was a trend-level effect of a positive association between reading times and knowledge gains under pretesting (*t*_(df = 130)_ = 1.866, *p* = 0.064, *partial η*^*2*^ = 0.03), while we found no significant associations under no-testing (t_(df = 122)_ = 0.612, *p* = 0.541) nor posttesting (*t*_(df = 142)_ = 0.093, *p* = 0.926).

## Discussion

Our findings demonstrate that practice testing enhances knowledge acquisition from persuasive texts but has limited effects on attitude change. Across two studies, both pretesting and posttesting improved memory for factual content compared to passive reading, confirming and extending prior research on the benefits of test-enhanced learning strategies in this context of persuasive texts^25,26,37^. Further, attitude systematically changed after reading the persuasive text (i.e., wolves were perceived as more positive and biodiversity loss was perceived as more threatening), but this change was not enhanced by adding pretesting or posttesting. Of note, however, we found a link between final knowledge and attitude for the pretesting condition only. While these results suggest that practice testing strengthens memory for persuasive content, they also highlight the complexity of the knowledge-attitude relationship and challenge the assumption that deeper cognitive engagement necessarily leads to belief revision.

This study extends prior research by exploring how practice testing interacts with persuasion, a domain where test-enhanced learning strategies have received little attention. We hypothesized that pretesting would promote deeper cognitive engagement by highlighting knowledge gaps, which, according to cognitive dissonance theory^17^, could increase motivation to resolve inconsistencies and lead to greater attitude change. While the expected overall effects of pretesting on attitude change were not observed, we did find a link between knowledge accuracy and attitude shifts in the pretesting group—an effect absent in the posttesting and no-testing conditions. This suggests that the way knowledge is acquired may influence how it relates to persuasion. When individuals generate responses before encountering correct information, they may engage more deeply with the material, making it more personally relevant and increasing the likelihood of aligning attitudes with newly acquired knowledge^12,26,38^.

The finding that knowledge accuracy was linked to attitude change in the pretesting condition raises important questions about the mechanisms underlying this relationship. One possibility is that pretesting increases the personal relevance of persuasive content for some individuals, prompting deeper engagement and greater shifts in both knowledge and attitude^12,22,26,38^. To clarify whether this association reflects the effect of pretesting itself or could be also explained by individual characteristics, we conducted control analyses. Neither working memory nor prior knowledge predicted attitude change in either study or accounted for the pretesting effect. In Study 1, the knowledge-attitude link remained, whereas in Study 2 it was initially marginal and became weaker once WM was included, suggesting a small influence of WM in reducing the association. Overall, these results suggest that the pretesting effect is not simply reducible to general cognitive ability or prior topic knowledge. At the same time, they highlight the need for future work to examine other potential moderators—such as cognitive motivation, openness to belief change, or sensitivity to demand characteristics—under more systematic control of possible confounds.

Most of the pretesting literature linking benefits to attentional processes has used designs without immediate feedback^12^, allowing the knowledge gap to persist into the study phase. We deliberately provided immediate feedback after each pretest item to avoid reinforcing misconceptions in a persuasive context. Unlike posttesting, pretesting requires generating responses before seeing the correct answer, which may increase self-involvement with the content and heighten engagement. Although immediate feedback may reduce the experience of prolonged cognitive dissonance, this generative process can still make corrections more salient and help strengthen the link between knowledge and attitude change. Future research should compare immediate and delayed or no-feedback pretesting to assess their relative effects on persuasion. Such work could also help clarify the factors contributing to the stronger knowledge–attitude link observed in pretesting.

Pretesting and posttesting led to similarly high knowledge gains for biodiversity loss (Study 1), whereas in Study 2, gains were slightly higher under posttesting. Our hypothesis that both methods would produce equivalent knowledge gains was therefore only partly confirmed. One possible explanation is that people in the pretesting group of Study 2 showed lower average working memory performance than those in Study 1 under both load conditions (Load 3: *M* = 0.70 < *M* = 0.75; Load 4: *M* = 0.57 < *M* = 0.64). Given evidence that working memory is related to testing success^39^, and possibly more so for pretesting^22^, this may have contributed to the weaker pretesting performance in Study 2.

These considerations are consistent with recent research that has revealed a complex relationship between cognitive abilities and the magnitude of pretesting effects^40,41^. In two within-subject experiments, Pan et al. (2025) found a “homogenization effect”: learners with lower fluid intelligence (Study 1) or lower WM capacity (Study 2) showed larger apparent benefits because they underperformed on read-only items but matched higher-ability peers on pretested items. However, these difference scores were unreliable, and analyses of absolute performance—particularly in Study 2 when all cognitive abilities were considered—showed that higher fluid intelligence consistently predicted better outcomes in the pretested condition, consistent with a “richer-get-richer” pattern.

In the present data, WM accuracy—measured via a task strongly correlated with fluid intelligence—predicted greater absolute knowledge gains in both pre- and posttesting groups, but not in the reading-only group. Cronin-Golomb et al. (2024), also focusing on absolute performance, showed that pretesting advantages vanished for productive integration tasks once verbal comprehension was controlled, but remained robust for fact recall. Since our outcome was fact recall, the pattern aligns with the view that pre- and posttesting amplify advantages for high-ability learners rather than equalize performance.

Together, these findings indicate that both learner abilities and task demands (e.g., recall vs. integration) interact with test format to determine who benefits most from pretesting. Future research should examine how individual differences shape not only knowledge acquisition but also attitudinal outcomes, and it should test whether WM functions as a mechanism—rather than an individual difference factor—through which pre- or posttesting and persuasion influence learning.

Moreover, this study provides an initial systematic test of practice testing effects on learning from persuasive texts. However, the specific pre- and posttesting variants we implemented may not have been optimally tailored to promote attitude change. Prior meta-analyses and empirical work suggest that factors such as question format, test alignment, and individual differences in cognitive abilities can moderate practice testing’s impact on both knowledge and persuasion^5,41^. Future research should therefore examine more cognitively demanding or mixed test formats and directly assess learner characteristics to determine how test-enhanced learning might be optimized for belief revision.

One limitation of this study is that it examined attitude change in a short-term context. It remains unclear whether test-enhanced learning might influence persuasion over longer retention intervals, as strengthened memory traces could shape reasoning and belief updating over time. Additionally, while we focused on persuasive texts related to environmental conservation, future studies should test whether similar effects occur with other types of persuasive content, especially those involving more deeply entrenched attitudes. Further, these experiments were conducted online, limiting control over participants’ environments and potential distractions. While validation checks were implemented, external influences remain a consideration. The study’s naturalistic design allowed for comparisons between testing conditions and passive reading but restricted mechanistic conclusions. Specifically, we proposed that pretesting heightens attention, linking knowledge to attitude change, yet the design did not allow direct testing of this assumption. Future research could use experimental psychophysiological methods to examine attentional engagement.

Overall, our findings underscore the distinction between knowledge retention and attitude change. While practice testing strategies enhances memory for persuasive content, they do not necessarily drive belief revision. These results contribute to a more nuanced understanding of how learning strategies interact with persuasion and suggest that pretesting may play a unique role in linking knowledge gains to attitude change under certain conditions. Further research is needed to clarify the cognitive mechanisms involved and to explore strategies that might more effectively integrate test-enhanced learning with persuasive influence.

### Study 1: methods

#### Participants

The study was conducted as an online-study in December 2020 in which participants were recruited via Prolific (https://app.prolific.co). Participants were paid 6 ₤, which was rated as a “Good” reward by the Prolific algorithm based on our estimated study completion time of 40 min. In addition, 3 ₤ of Prolific service fee were paid per participant. Because we aimed to test a sample that represents the general population, participants of a broad age range between 22 and 67 years (50% male) and from all levels of socio-economic status (SES) were included into the study. Participants were recruited worldwide, with the majority (78.8%) of all participants living in the UK. Ethics approval for Study 1 and 2 was obtained from the ethics committee of DIPF | Leibniz Institute for Research and Information in Education in Frankfurt, Germany. All methods were carried out in accordance with relevant guidelines and regulations. Informed consent was obtained from all participants in accordance with the approved protocol.

To reach a total sample size of approximately *N* = 420 (3 × 140) based on statistical power considerations (cf., Data Analysis), we tested 500 people in total (estimating that ~ 15% of participants will provide unusable data as specified below). The final sample consisted of *N* = 454 participants aged 20 to 65 years (229 male, 225 female; *M* = 36.34 years, *SD* = 11.70). Self-reported SES ranged from 1 to 10 on a rating scale between 1 (extreme low) and 10 (extreme high) (*M* = 5.54, *SD* = 1.55). Most of the participants (*n* = 347) reported United Kingdom as their Nationality.

The pre-testing group consisted of *n* = 155 participants aged between 20 and 64 years (70 male, 85 female; *M* = 36.55 years, *SD* = 11.81). Self-reported SES in this group ranged from 2 to 10 (*M* = 5.56, *SD* = 1.55). The post-testing group consisted of *n* = 161 participants aged between 20 and 65 years (85 male, 76 female; *M* = 36.02 years, *SD* = 11.49). Self-reported SES in this group ranged from 1 to 8 (*M* = 5.76, *SD* = 1.46). The no-testing group consisted of *n* = 138 participants aged between 20 and 65 years (74 male, 64 female; *M* = 36.48 years, *SD* = 11.91). Self-reported SES in this group ranged from 2 to 10 (*M* = 5.23, *SD* = 1.63).

#### Procedure

We created the experiment using PsychoPy builder^42^ allowing the online-testing. We made the study available for online distribution by uploading the java script files to Pavlovia (https://pavlovia.org). All experimental and log files were stored at the GitLab repository within Pavlovia. The activated study link provided by Pavlovia was then connected to the Prolific participant recruitment service platform.

Before the experiment started, a welcome screen instructed the participants to complete the study alone in front of their computer or lap top using a keyboard and mouse device within one hour; when needed a break between the individual parts of the study could be taken. All other browser windows or programs should be closed. This was followed by another screen showing a general instruction which explained that the study consisted of six short parts in total, and that each part lasted between 2 and 10 min. In addition, it was mentioned that there will be some practice trials in each study part before the real experiment starts. Participants were motivated to pay full attention and to try to respond as accurately as possible. Before each study part, it was informed about the task and how it should be responded, i.e., by using the mouse device and number keys and the space bar to continue with the next trial or task.

#### Design

The experimental design comprised one between-subject factor (i.e., study group) with three levels (1 = no-testing, 2 = posttesting, 3 = pretesting). Participants were randomly assigned to one of three different groups.

The experiment consisted of the following six parts that varied in order depending on the study group: (a) attitude questions towards extinction before a learning session; (b) an interim knowledge test in which participants were prompted to make a prediction about a numerical fact regarding extinction (e.g., “x % of amphibians are at risk of extinction”) before the correct number was revealed. Here they were not explicitly instructed to memorize tested items for later recall. Feedback was provided immediately after each response, ensuring exposure to the correct information; (c) a text reading part in which participants were asked to read a text on the severity of loss of biodiversity; (d) a numerical working memory (WM) updating task; which was included as a filler task and to ensure that individuals did not systematically differ in their cognitive capacities between study groups, (e) attitude items towards extinction that were the same questions as in (a) but after the learning session; (f) a final knowledge test consisting of the same items as in the interim knowledge test, but without presentation of the correct response in each trial.

In the pretesting condition, participants first completed the initial knowledge test (answering numerical fact-based questions) with immediate corrective feedback on each item (b), and then read the persuasive text (c). In the posttesting condition, participants read the persuasive text (c), then answered the same knowledge questions with feedback (b). In the no-testing control, participants read the text (c) without any knowledge test before or after reading. All participants completed attitude measures before (a) and after reading (e), a working memory task (d), and a final knowledge test (f). What tasks were performed by which study group, and in what order, is summarized in Table 1.

#### Tasks and measurements

##### Knowledge

Knowledge was measured twice as mean response accuracy (%) for fourteen items on numerical facts presented in the text. In the ‘interim knowledge test’ participants received feedback (i.e., display of correct answers) while no feedback was presented in the ‘final knowledge test’. The interim and final knowledge task consisted of the same fourteen items (see full list of 14 items in Supplement Information S1). After the instruction screen and two practice trials, participants were presented with the experimental trials, whereby each trial consisted of one item, i.e. a question, e.g., “How many animal and plant species are currently threatened with extinction?”. Below this question, an empty field was displayed in which participants were asked to type their answer and then press enter to see the correct answer (feedback was only displayed for the interim knowledge task).

##### Text reading part

Participants were presented with a text on recent findings concerning changes in biodiversity. The text was divided into sections on thirteen screens with a similar number of words. A text section was presented for at least 4 s on the screen, after this time participants could press space to continue with the next section. Participants were instructed to read the text carefully and were informed that they will be asked some questions about the content of the text afterwards.

##### Attitude towards extinction

Attitude towards extinction was also measured twice: before (‘pre-attitude’) and after the experiment (‘post-attitude’). Participants had to answer the following questions on a 10-point rating scale ranging from 1 (“not at all”) to 10 (“extremely”): “How threatening do you think the reduction in biodiversity is for your living conditions?”, “Is the reduction in biodiversity something you worry about?”, and “Do you think that you will feel the impact of the reduction in biodiversity in your life?”. Each question was presented on a separate screen; below a question, the rating scale was displayed in form of a horizontal bar with 10 gradations, and participants responded by moving a triangle marker along this scale with the mouse. The mean score across responses from all items was computed for each condition (pre-/post-attitude) and participant. The change in attitude was scored as the change in attitude mean score: $\Delta$ attitude = post-attitude—pre-attitude.

##### Working memory updating

WM performance was assessed using a numerical WM updating task in which participants memorized and updated several one-digit numbers [cf., 43]. During encoding, three or four single-digit numbers (between 0—9), corresponding to load 3 and load 4 conditions, were presented simultaneously for 3000 ms, each on one of three or four cells arranged around the center of the display. Then a sequence of four or five updating operations were presented for load 3 and load 4 conditions, respectively. The updating operations were additions and subtractions from + 8 to -8 and were applied to the memorized digits (1500 ms presentation time per operation with an inter-stimulus-interval/ISI of 250 ms). At the end of each trial, participants were prompted to enter the end results for each item. After they confirmed their responses, a feedback followed by showing the correct answers. Nine trials per load condition were presented preceded by three training trials with two items (load 2) and two further training trials of loads 3 and 4.

### Data analysis

Data were analyzed with linear mixed effects models using the *nlme* package^44^ in R (https://www.r-project.org, R Core Team, 2016). Before modeling, data were cleaned as specified in pre-registration (https://osf.io/a4ghd). To all models, significance of fixed effects was evaluated using the package’s default estimation of degrees of freedom. A conventional alpha level of 0.05 was applied to all tests. Random effects were allowed to co-vary freely (i.e., unstructured G-matrix).

Preregistration note: Analyses of the primary outcomes (final knowledge accuracy and attitude change) were preregistered prior to data collection (https://osf.io/a4ghd). The working memory updating task was also preregistered under “Other” as a measure to (1) clear information from WM before the memory test and (2) explore whether WM moderated group effects. Other analyses (e.g., reading time, additional moderation tests) were conducted exploratorily and are labeled as such in the Results.

To determine the sample size, we conducted a statistical power analysis using the R package *Superpower*^45^. A design was specified comprising one between-subject factor (i.e., study group) with three levels/conditions, with n = 140 participants per condition. Expected effect sizes were informed by results from two pilot studies which followed the same procedure and used the same design and items as the present study, along with three additional attitude questions. As in the main studies, participants were recruited via Prolific with a final sample size of n = 35 in the pretesting group, n = 53 in the posttesting group, and n = 28 in the no-testing group. The results of the pilot studies indicated significant differences in final knowledge outcomes, specifically with pretesting/posttesting > no-testing, but no significant difference between the two testing conditions. An ordinal interaction was also observed in attitude change across study groups, showing the pattern: Pretesting > Posttesting > No-testing. Based on these design specifications, exact power estimates for the effects of study group on final knowledge and attitude change were computed through two separate power simulations, each assuming an alpha level of 0.05. For the Bonferroni-adjusted pairwise comparisons of knowledge effects, results revealed the following estimates: *power* = 99.99%, partial η^2^ = 0.13 (Pretesting > No-testing); *power* = 99.99%, partial η^2^ = 0.12 (Posttesting > No-testing); and *power* = 2.79%, partial η^2^ = 0.00 (Pretesting ~ Posttesting). For one-sided contrasts of the attitude effects, estimates were *power* = 86.40%, partial η^2^ = 0.018 (Pretesting > Posttesting) and *power* = 77.86%, partial η^2^ = 0.014 (Posttesting > No-testing). Given these results and considering that effect sizes should be interpreted within the specific context of a study^46^, we decided to select a sample size of n = 140 per study group to detect a meaningful effect.

For hypotheses H1a-c, we tested whether response accuracy in the final knowledge test depended on study group based on the following comparisons: Posttesting > No-testing, Pretesting > No-testing and Pretesting ~ Posttesting (Model H1). For hypotheses H2a-b, we tested whether the change in attitude from pre to post (i.e., delta attitude) was predicted by study group using a priori contrast coding^47^: Posttesting > No-testing, Pretesting > Posttesting (Model H2). Both models included a random intercept for Subject (see preregistered hypotheses at https://osf.io/a4ghd). Note that what is described here as the pretesting condition was referred to as ‘Prediction’ in the preregistration, and the no-testing condition was labeled ‘Read-only’.

To support non-significant analyses of our core hypotheses, we employed an equivalent Bayesian analysis using the *brms* package^48^ in which default weakly informative priors were applied to all parameters. The model estimation was based on four chains of 2,000 iterations each, including a burn-in of 500 iterations, resulting in 6,000 posterior samples.

In addition, we examined whether a participants’ attitude towards the loss in biodiversity (i.e., mean rating response across items) changed from pre to post measurement occasion (pre-/post-attitude, coded as factor) and/or differed between study groups. We firstly tested for main effects of pre-/post-attitude and study condition (Model 1a), and then, in a second model, we tested for interactions among these two predictor variables (Model 1b). In both models, a random intercept for Subject and a random slope effect for the effect of pre-/post-attitude were estimated. Pairwise comparisons for study group (coded as factor) were evaluated by computing contrasts of estimated marginal means.

Further, we explored links between knowledge and attitude using random intercept models separately for each study group in which post-attitude rating was predicted by response accuracy in the final knowledge test (No-testing: Model 2a; Posttesting: Model 2b, Pretesting: Model 2c).

We conducted several control analyses. As a manipulation check, we first tested whether participants under pretesting differed from participants under posttesting in their interim knowledge using a model with response accuracy in the interim knowledge test as dependent variable and study group as predictor. Further, we tested whether working memory performance (i.e., mean accuracy) was predicted by load (i.e., load 3 and 4; coded as factor) and study group. We firstly tested for main effects of these two predictors, and then in second model, we also tested for their interaction. Both models included a random effect for load. To ensure that study groups showed similar baseline response ratings, we estimated the effect of study group on the rating of the pre-attitude questions. Moreover, we tested whether study groups differed in reading times with a model in which reading duration (i.e., total time/ accumulated time spent to each page) was predicted by study group, and whether reading duration was linked to final knowledge within each study group. For all models of these control analyses, a random intercept for Subject was estimated.

### Study 2: methods

We used the same methodological approach as in Study 1 but a different persuasive text including facts on “wolf populations”. We aimed to conceptually replicate the findings on knowledge gains, and to test whether changes in attitude change depend on testing when a different, more controversial topic was presented.

#### Participants

The second study was also conducted as an online-study (in July 2022) using the same platforms, compensation and inclusion criteria as in the first study (22 and 67 years, 50% male, all levels of socio-economic status). Participants were recruited from the US and the UK and their first language was English.

We tested 450 people in total (estimating that ~ 10% of participants will provide unusable data). A total of 50 participants needed to be excluded due to incomplete responses or failed attention checks. The final sample consisted of *N* = 400 participants in total (pre-testing: *n* = 132; post-testing: *n* = 144; no-testing: *n* = 124). Participants were aged between 21 and 66 years (197 male, 197 female, 6 no response; *M* = 36.61 years, *SD* = 12.17). Self-reported SES ranged from 1 to 10 on a rating scale between 1 (extreme low) and 10 (extreme high) (*M* = 5.33, *SD* = 1.65). Most of the participants (*n* = 33) reported United Kingdom as their Nationality.

The pre-testing group consisted of *n* = 132 participants aged between 21 and 66 years (59 male, 69 female, 4 no response; *M* = 38.41 years, *SD* = 11.83). Self-reported SES in this group ranged from 2 to 10 (*M* = 5.16, *SD* = 1.59). The post-testing group consisted of *n* = 144 participants aged between 21 and 66 years (72 male, 70 female, 2 no response; *M* = 40.35 years, *SD* = 12.64). Self-reported SES in this group ranged from 1 to 10 (*M* = 5.49, *SD* = 1.60). The no-testing group consisted of *n* = 124 participants aged between 21 and 66 years (66 male, 58 female, 2 no response; *M* = 36.84 years, *SD* = 11.80). Self-reported SES in this group ranged from 2 to 10 (*M* = 5.31, *SD* = 1.76).

#### Procedure and design

In contrast to Study 1, we presented attitude questions and knowledge items regarding wolves (e.g., “How many wolf packs does Germany’s wolf population comprise today? About…”) (see full list of knowledge items in Supplement Information S2). In addition, we included an item asking about participants’ trust in the persuasive text i.e., “How trustworthy did you find the article?”.

#### Measurements

##### Knowledge

Knowledge was measured in the same way as in Study 1.

##### Attitude towards wolves

Participants had to answer the following questions on a 7-point bidirectional rating scale ranging from 1 (“extremely negative”) to 7 (“extremely positive”): 0 “What is your attitude towards wolves in general?”, 1 “What impact do you think it would have on the environment if wolves were to settle in your county?”, 2 “What impact do you think it would have on the people in your county if wolves were to settle there?”, 3 “How do you think your life would be impacted if wolves established in your county?”. Item 0 was tested for the purpose of another study and was not included in the analyses of the present paper. As in Study 1, the mean score across responses from items 1 to 3 was computed for each condition (pre-/post-attitude) and participant. The change in attitude was scored as $\Delta$ attitude = post-attitude—pre-attitude.

##### Data analysis

Data were prepared using the same criteria as in Study 1. Here, participants with a “pre-attitude” mean score of 7 (corresponding to the highest value) were excluded. We also applied the same analyses as in Study 1.

## Supplementary Information

Below is the link to the electronic supplementary material.

Supplementary Material 1

## Data Availability

The study’s hypotheses (H1a-c and H2a-b) were preregistered on OSF (https://osf.io/a4ghd). All data and analysis scripts are publicly available on OSF and can be accessed at https://osf.io/gfr69/. This study complies with the Transparency and Openness Promotion Guidelines by ensuring accessibility of materials and preregistration information.
