# Supplementary material for: Practice testing enhances learning but not attitude change from persuasive texts
Source: Sci Rep. 2025 Sep 25;15:32935. doi: 10.1038/s41598-025-20874-1 (PMC12464326; doi:10.1038/s41598-025-20874-1)
Supplement: Supplementary file 1 — Supplementary Material 1 [file 41598_2025_20874_MOESM1_ESM.pdf]

Supplementary Information

Title: Practice Testing Enhances Learning but Not Attitude Change from Persuasive Texts

Authors: Elena M. Galeano Weber, Lisa Lehnen, Doug Lombardi, & Garvin Brod

Study 1

S1. Knowledge Items

1. How many animal and plant species are currently threatened with extinction?
2. By how much has the world's population grown since 1970?
3. How many hectares of tropical forest were lost between 1980 and 2000?
4. What percentage of wetland present in 1700 was still in existence in 2000?
5. By how much have urban areas expanded since 1992?
6. What percentage of animals and plants are currently threatened with extinction?
7. What percentage of amphibians are at risk of extinction according to the IUCN Red List?
8. What percentage of birds are at risk of extinction according to the IUCN Red List?
9. By how much percent has the productivity of the land surface of the Earth reduced because of degrading soils?
10. How many tonnes of heavy metals, solvents and other wastes are dumped in the world's waters every year?
11. What percentage of the world's oceans were free from human pressure (in 2014)?
12. What percentage of fish stocks are harvested at unsustainable levels?
13. By what percentage has coral cover on reefs approximately reduced over the past 150 years?
14. What percentage of agriculture is related to meat production?

Study 2

S2. Knowledge Items

1. How many wolf packs does Germany's wolf population comprise today? About…
2. The German wolf population covers what percentage of the country? About…
3. When did wolves go extinct in Denmark?
4. For how many wolves did France approve culling after protests in 2017? Up to…
5. How many wolves remained of the Finnish wolf population after culling?
6. What percentage of the Norwegian wolf population was slaughtered in winter 2017/18? About…
7. What percentage of the German population find it important to protect wild wolf populations for future generations? About…
8. How much has the number of deer-vehicle collisions decreased in Wisconsin after wolves established there? Nearly…
9. How much greater are the economic benefits of wolves in Wisconsin compared to their economic costs? More than…
10. How much did animal tuberculosis in wild boar in Asturias (Spain) decrease during the time of wolf presence? More than…
11. What percentage of the German population find that the presence of wild wolves increases the value of a landscape? About…
12. What percentage of the German population think that wolves should be allowed to live in Germany ? About…
13. How many wolves live on the Iberian Peninsula?
14. What is the maximum number of wolves accepted in Norway, according to Guillaume Chapron ?
